# Hepatic cell mobilization for protection against ischemic myocardial injury

**DOI:** 10.1038/s41598-021-94170-z

**Published:** 2021-08-04

**Authors:** Shu Q. Liu, John B. Troy, Chi-Hao Luan, Roger J. Guillory

**Affiliations:** 1 Biomedical Engineering Department, Northwestern University, Evanston, IL 60208 USA; 2 High Throughput Analysis Laboratory, Northwestern University, Evanston, IL 60208 USA; 3 Present Address: Department of Biomedical Engineering, Michigan Technological University, Houghton, MI 49931 USA

**Keywords:** Physiology, Cardiology

## Abstract

The heart is capable of activating protective mechanisms in response to ischemic injury to support myocardial survival and performance. These mechanisms have been recognized primarily in the ischemic heart, involving paracrine signaling processes. Here, we report a distant cardioprotective mechanism involving hepatic cell mobilization to the ischemic myocardium in response to experimental myocardial ischemia–reperfusion (MI-R) injury. A parabiotic mouse model was generated by surgical skin-union of two mice and used to induce bilateral MI-R injury with unilateral hepatectomy, establishing concurrent gain- and loss-of-hepatic cell mobilization conditions. Hepatic cells, identified based on the cell-specific expression of enhanced YFP, were found in the ischemic myocardium of parabiotic mice with intact liver (0.2 ± 0.1%, 1.1 ± 0.3%, 2.7 ± 0.6, and 0.7 ± 0.4% at 1, 3, 5, and 10 days, respectively, in reference to the total cell nuclei), but not significantly in the ischemic myocardium of parabiotic mice with hepatectomy (0 ± 0%, 0.1 ± 0.1%, 0.3 ± 0.2%, and 0.08 ± 0.08% at the same time points). The mobilized hepatic cells were able to express and release trefoil factor 3 (TFF3), a protein mitigating MI-R injury as demonstrated in TFF3^−/−^ mice (myocardium infarcts 17.6 ± 2.3%, 20.7 ± 2.6%, and 15.3 ± 3.8% at 1, 5, and 10 days, respectively) in reference to wildtype mice (11.7 ± 1.9%, 13.8 ± 2.3%, and 11.0 ± 1.8% at the same time points). These observations suggest that MI-R injury can induce hepatic cell mobilization to support myocardial survival by releasing TFF3.

## Introduction

Ischemic myocardial injury can activate cardioprotective mechanisms to support cardiomyocyte survival and mitigate myocardial infarction^1–8^. The biological basis of these cardioprotective mechanisms is the cell-survival supporting systems. These systems have possibly developed through evolution in response to environmental insults, such as physical, chemical, and microbiological injuries^8^. To date, most cardioprotective systems have been found within the heart, involving various signaling pathways that can be activated by ischemic cell-released paracrine factors such as adenosine^9–11^, bradykinin^12,13^, opioids^14–16^, and/or growth-regulatory factors^17–21^. In this report, we demonstrate a distant cardioprotective mechanism involving the liver, an organ capable of discharging its cells to the circulatory system in response to experimental myocardial ischemia–reperfusion (MI-R) injury^22^. The discharged hepatic cells were able to mobilize to the ischemic myocardium to exert a protective action by releasing trefoil factor 3 (TFF3), an endocrine protein known to support cardiomyocyte survival^23^. The significance of hepatic cell mobilization is to support the paracrine cardioprotective mechanisms, thereby augmenting cardioprotection.

Hepatic cell mobilization is an acute, transient process occurring after ischemic myocardial injury^22^. Given its relationship with myocardial ischemia, hepatic cell mobilization has been considered a process contributing to cardioprotection^22^. However, it has been difficult to confirm its cardioprotective role because of difficulties in establishing a loss-of-hepatic cell mobilization condition in an animal model. An effective approach is to remove the liver of an animal to eliminate hepatic cell mobilization; however, this approach causes rapid animal death. To overcome such a problem, a parabiotic mouse model (surgical skin-union of two mice) was developed and used to induce MI-R injury in both mice while the liver was removed in one mouse, providing a gain-of-hepatic cell mobilization condition in the mouse with intact liver and a concurrent loss-of-hepatic cell mobilization condition in the mouse with hepatectomy. As the mobilized hepatic cells in the mouse with the intact liver were not able to reach the mouse with hepatectomy, this model was used to evaluate the cardioprotective role of the mobilized hepatic cells by analyzing experimental outcomes in the presence and absence of hepatic cells in the ischemic myocardium.

A fundamental question is how the mobilized hepatic cells contribute to cardioprotection in MI-R injury. One potential mechanism is to release cardioprotective factors directly from the hepatic cells recruited to the ischemic myocardium, an approach conceivably more effective than distant release from the liver. The liver has been shown to upregulate and release the endocrine factor TFF3^23^, a protein originally identified in intestinal epithelial cells and recognized as a protective factor against endothelial injury under physiological and pathological conditions^24–29^. Ischemic myocardial injury has been shown to stimulate the expression of TFF3 in the hepatic cells^23^. TFF3 was able to mitigate ischemic myocardial injury in a mouse model with recombinant TFF3 administration^23^. However, the cardioprotective role of TFF3 has not been confirmed by using a genetically modified model. The present investigation was designed to demonstrate the mechanisms by which the mobilized hepatic cells were able to protect the ischemic myocardium by releasing TFF3 and confirm the cardioprotective role of TFF3 by using a TFF3^−/−^ mouse model with and without recombinant TFF3 administration.

## Results

### Mobilization of hepatic cells to the circulatory system and ischemic myocardium

**Figure 1:**
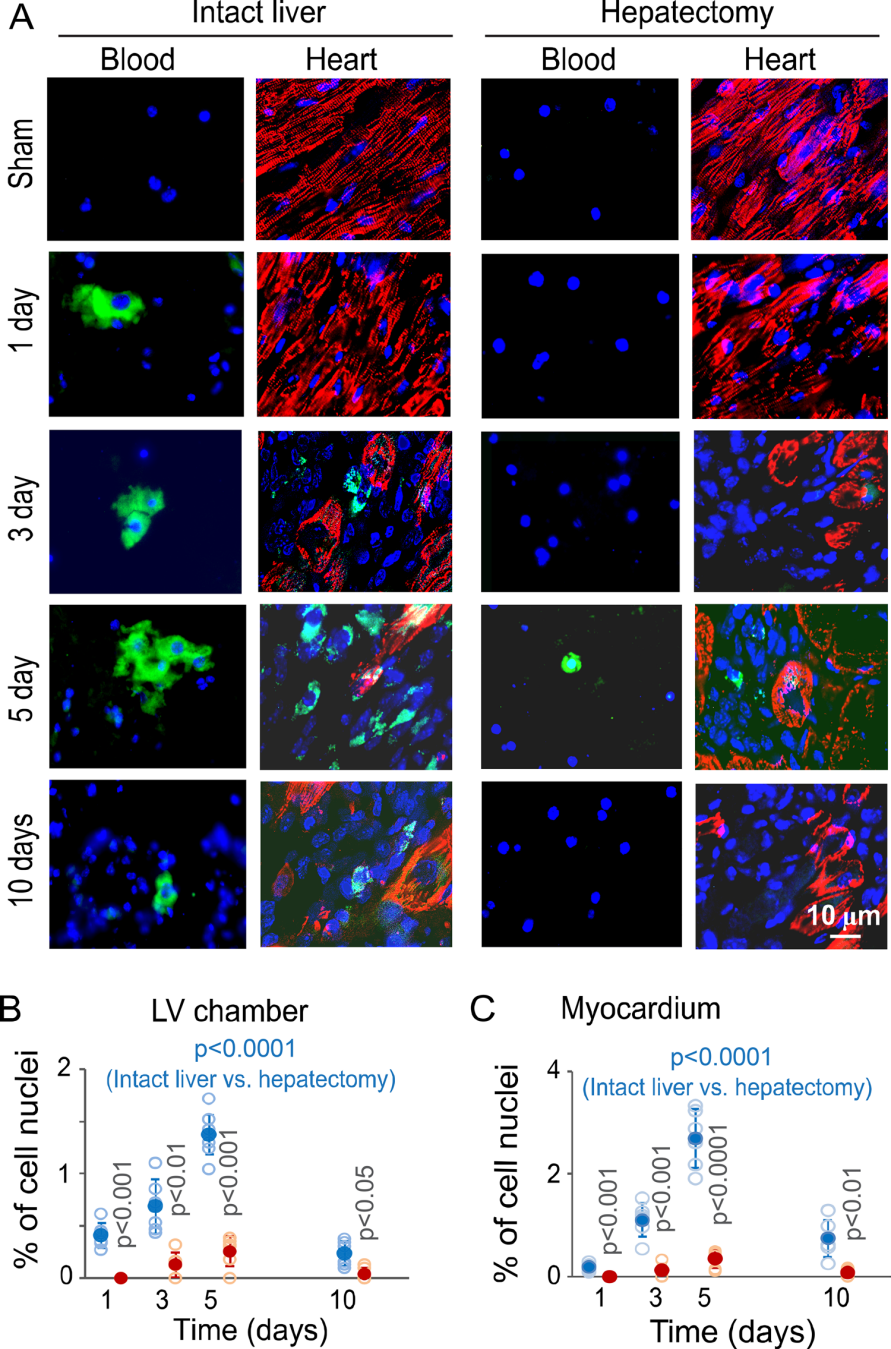
Mobilization of hepatic cells to the circulatory system and ischemic myocardium in response to MI-R injury. (**A**) eYFP-positive cells from left ventricular blood samples and the ischemic myocardium of liver-intact and hepatectomized parabiotic Alb-Cre/eYFP mice. Green: eYFP. Red: Antibody-labeled cardiac troponin I. Blue: Hoechst 33258-stained cell nuclei. The length scale is for all panels. (**B**, **C**) Graphic representations of eYFP-positive cell populations in the left ventricular chamber (**B**) and ischemic myocardium (**C**) of liver-intact and hepatectomized parabiotic Alb-Cre/eYFP mice. The % of eYFP-positive cells was calculated in reference to the total cell nuclei. Solid blue and red circles: Means ± SDs from liver-intact and hepatectomized Alb-Cre/eYFP mice with MI-R injury, respectively. Open blue and red circles: Raw data points (n = 6) from liver-intact and hepatectomized Alb-Cre/eYFP mice with MI-R injury, respectively. The blue colored p values are for comparisons between the liver-intact and hepatectomized parabiotic mice by ANOVA. The p value on each red-colored column is for a post-hoc comparison between the liver-intact and hepatectomized parabiotic mice at each time point.

Hepatic cells were mobilized to the circulatory system in response to MI-R injury, as demonstrated by using the mouse model expressing hepatic cell-specific eYFP (enhanced yellow fluorescent protein)^22^. Circulating eYFP-positive hepatic cells were found in the left ventricular chamber at 1 day, reached a peak population at 5 days, and returned toward the sham control level at 10 days after MI-R injury (Fig. 1A,B). The mobilized eYFP-positive hepatic cells were recruited to the ischemic, but not the intact and sham-control, myocardium with a peak population at 5 days after MI-R injury as detected by fluorescence microscopy (Fig. 1A,C; supplementary Fig. 1 and Table 1).

**Figure 2:**
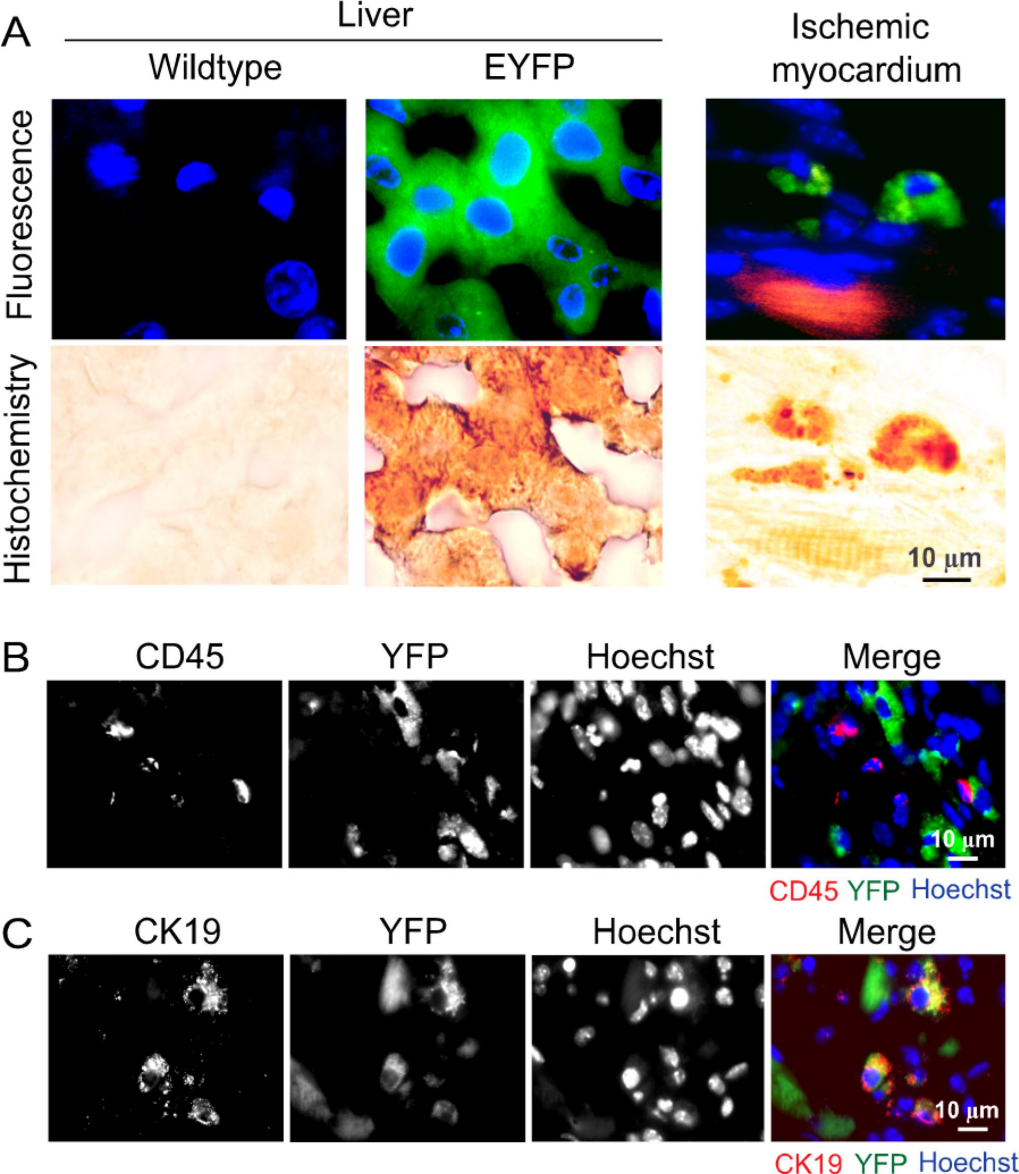
Characterization of hepatic cells in the liver and ischemic myocardium. (**A**) Confirmation of eYFP-positive hepatic cells in the liver and ischemic myocardium of Alb-Cre/eYFP mice with 5-day MI-R injury by fluorescence and immunohistochemistry microscopy. Each pair of fluorescence and immunohistochemistry images was from the same specimen. Green: eYFP. Red: Antibody-labeled cardiac troponin I. Blue: Hoechst 33,258-stained cell nuclei. Brown: Antibody-labeled eYFP by immunohistochemistry. The length scale is for all panels. (**B**, **C**) Fluorescence micrographs showing eYFP-positive cells, CD45-positive cells, and cytokeratin 19 (CK19)-positive cells in the ischemic myocardium of an Alb-Cre/eYFP mouse with 5-day MI-R injury. Note co-localization of eYFP with CK19, suggesting the presence of bile ductular epithelial cells.

The presence of eYFP-positive hepatic cells in the ischemic myocardium was confirmed by immunohistochemistry with an anti-eYFP antibody (Fig. 2A). The ischemic myocardium-recruited eYFP-positive cells did not express the leukocyte marker CD45 (Fig. 2B). Unilateral hepatectomy in parabiotic mice did not significantly influence leukocyte recruitment to the ischemic myocardium (supplementary Table 2). Selected eYFP-positive cells (23 ± 9% in reference to the total eYFP-positive cells) in the ischemic myocardium expressed cytokeratin 19, a protein marker used for identifying hepatic biliary epithelial cells in the liver (Fig. 2C, supplementary Table 3). These observations suggest that hepatocytes and biliary epithelial cells can be mobilized to the circulatory system and ischemic myocardium in response to MI-R injury.

The parabiotic mouse model with bilateral MI-R injury and unilateral hepatectomy demonstrated uneven distributions of the mobilized eYFP-positive hepatic cells in the circulatory system and ischemic myocardium between the liver-intact and hepatectomized parabiotic mice. Whereas hepatic cells were present in the left ventricular chamber and ischemic myocardium of the liver-intact parabiotic mice in MI-R injury, these cells were found at significantly lower levels at the same locations of the hepatectomized parabiotic mice at the same time points (Fig. 1; supplementary Table 1). There was no evidence showing exchanges in mobilized hepatic cells between the two mice of a parabiotic pair, as shown in a parabiotic model with paired Alb-Cre/eYFP and C57BL/6J mice with bilateral MI-R injury and alternated unilateral hepatectomy (supplementary Fig. 2, supplementary Table 4). These observations suggest that the mobilized hepatic cells from the liver-intact mouse were not able to reach the hepatectomized mouse in a parabiotic pair. Thus, this parabiotic mouse model can be used to establish concurrent gain- and loss-of-hepatic cell conditions in the ischemic myocardium, allowing the evaluation of the role of the mobilized hepatic cells in cardioprotection in MI-R injury.

### The fate of the mobilized hepatic cells in the circulatory system

The mobilized eYFP-positive hepatic cells in MI-R injury were found in the left ventricular chamber (Fig. 1) and ascending aorta (supplementary Fig. 3), but not significantly in the distant peripheral arteries and veins, including the femoral artery and vein (supplementary Fig. 3). The circulating eYFP-positive hepatic cell population in the ascending aorta was about 10 times larger than that in the femoral artery and vein (supplementary Fig. 3). These observations suggested that the circulating hepatic cells were eliminated within the aorta. However, the underlying mechanisms remain to be tested.

### Role of the mobilized hepatic cells in the mitigation of MI-R injury

**Figure 3:**
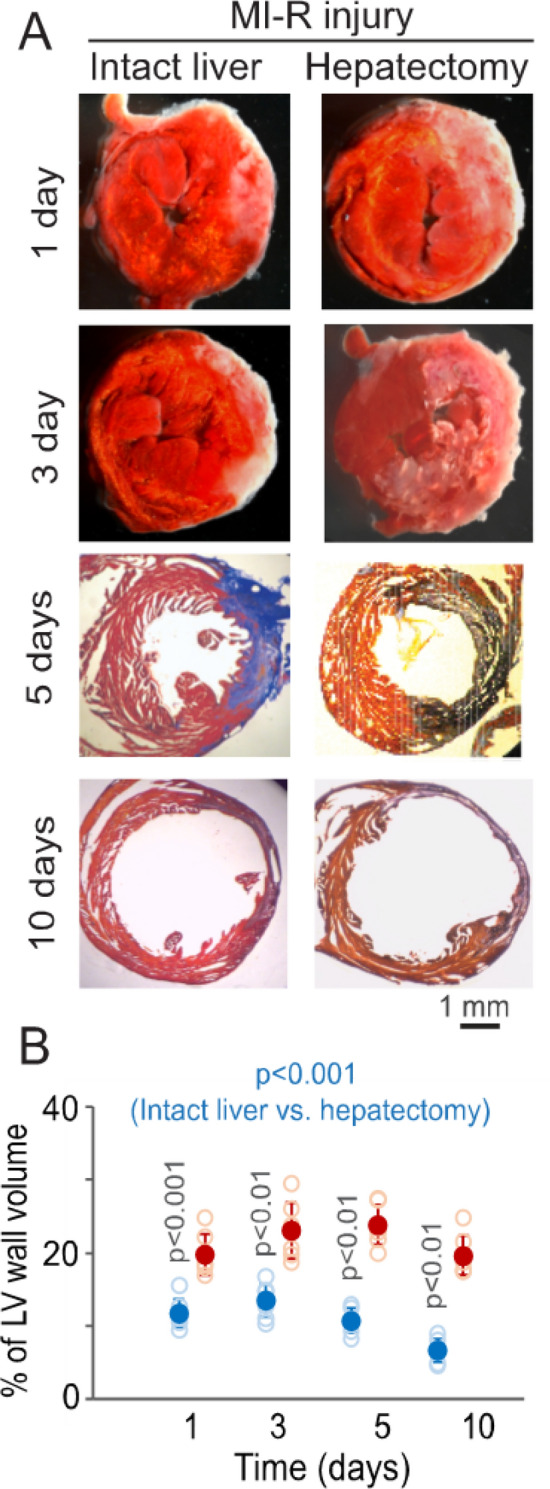
Myocardial infarction in liver-intact and hepatectomized parabiotic mice. (**A**) Left ventricular specimens from liver-intact and hepatectomized parabiotic mice, showing TTC-stained myocardial infarcts (pale) at 1 and 3 days and AZAN-stained myocardial infarcts/fibrotic tissue (blue) at 5 and 10 days after MI-R injury. Red: Intact myocardium. The length scale is for all panels. (**B**) Graphic representation of the % of myocardial infarcts in reference to the total volume of the left ventricular wall. Solid blue and red circles: Means ± SDs from liver-intact and hepatectomized parabiotic mice with MI-R injury, respectively. Open blue and red circles: Raw data points (n = 6) from liver-intact and hepatectomized parabiotic mice with MI-R injury, respectively. The blue-colored p value is for comparison between the liver-intact and hepatectomized mice by ANOVA. The p value on each blue-colored column is for post-hoc comparison between the liver-intact and hepatectomized mice at each time point.

**Figure 4:**
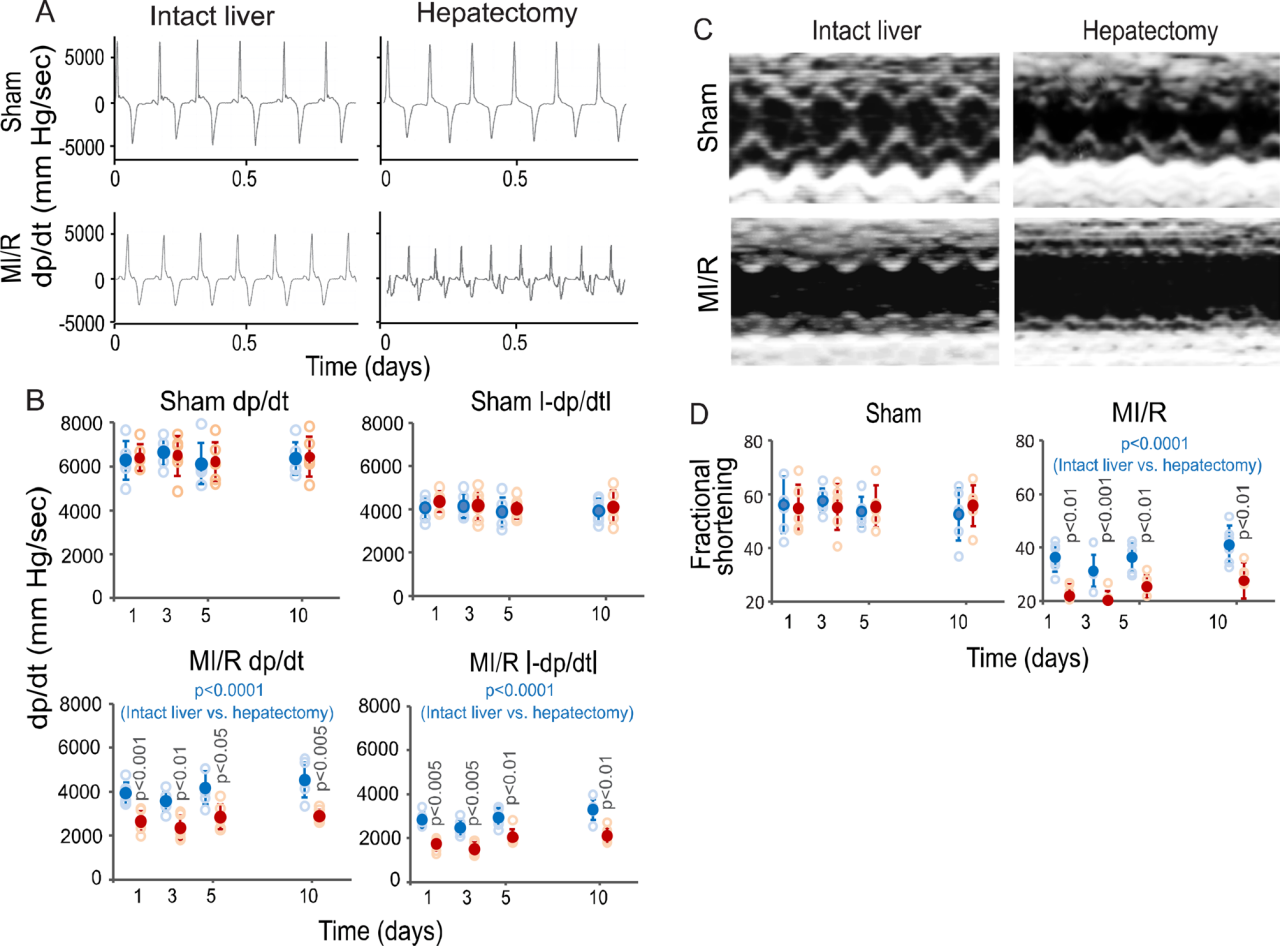
Left ventricular (LV) performance analyses in MI-R injury. (**A**) LV dp/dt recorded from liver-intact and hepatectomized parabiotic mice with cardiac sham operation and MI-R injury at 5 days. (**B**) Graphic representations of LV dp/dt and l-dp/dtl (absolute -dp/dt) from liver-intact and hepatectomized parabiotic mice with cardiac sham operation and MI-R injury. Solid blue and red circles: Means ± SDs from liver-intact and hepatectomized parabiotic mice with MI-R injury, respectively. Open blue and red circles: Raw data points (n = 6) from liver-intact and hepatectomized parabiotic mice with MI-R injury, respectively. The blue-colored p values are for comparisons between the liver-intact and hepatectomized parabiotic mice by ANOVA. The p value on each red-colored column is for post hoc comparison between the liver-intact and hepatectomized parabiotic mice at each time point. (**C**) LV echocardiograms from liver-intact and hepatectomized parabiotic mice with cardiac sham operation and MI-R injury at 5 days. (**D**) Graphic representations of LV fractional shortenings from liver-intact and hepatectomized parabiotic mice with cardiac sham operation and MI/R injury. Solid blue and red circles: Means ± SDs from liver-intact and hepatectomized parabiotic mice with MI-R injury, respectively. Open blue and red circles: Raw data points (n = 6) from liver-intact and hepatectomized parabiotic mice with MI-R injury, respectively. The blue-colored p value is for comparison between the liver-intact and hepatectomized parabiotic mice by ANOVA. The p value on each red-colored column is for comparison between the liver-intact and hepatectomized parabiotic mice at each time point.

The parabiotic mouse model was used to test the role of the mobilized hepatic cells in the mitigation of MI-R injury. In parabiotic mouse pairs with bilateral MI-R injury and unilateral hepatectomy, the liver-intact parabiotic mice with mobilized hepatic cells showed significantly reduced myocardial infarction compared with hepatectomized parabiotic mice at all observation times (Fig. 3). Cardiac sham operation did not cause myocardial infarction in liver-intact and hepatectomized mice at selected time points (1 and 5 days) (supplementary Fig. 4). Functional analyses showed that the levels of left ventricular dp/dt (Fig. 4A,B) and fractional shortening (Fig. 4C,D) were significantly higher in the liver-intact parabiotic mice than the levels of the two parameters in the hepatectomized parabiotic mice in MI-R injury. However, the left ventricular wall thickness was not significantly different between the liver-intact and hepatectomized mice (Supplementary Fig. 5). These observations suggest that the mobilized hepatic cells support myocardial survival and performance in MI-R injury.

### Hepatic cell transplantation for myocardial protection

**Figure 5:**
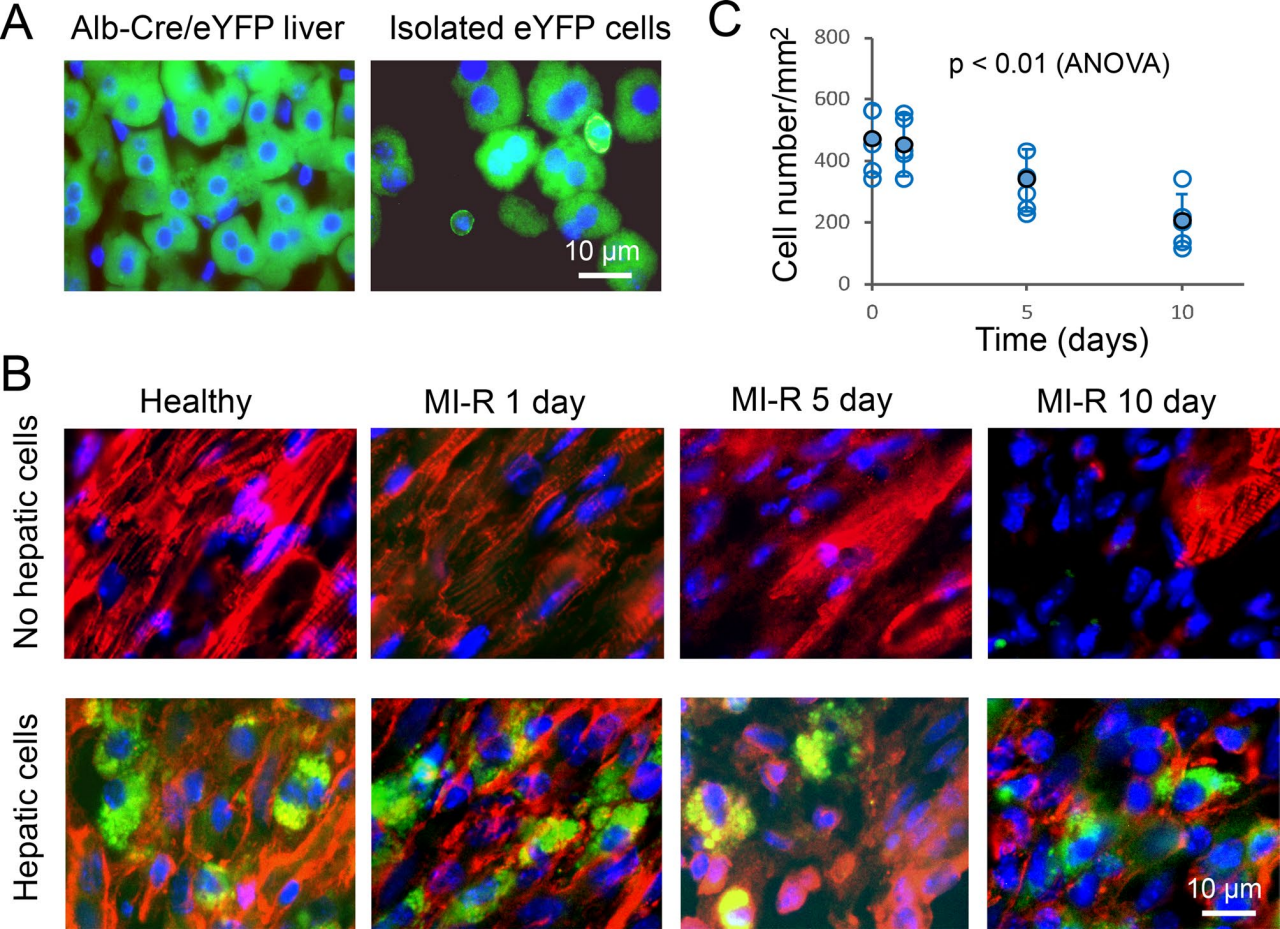
eYFP-positive hepatic cells transplanted to the myocardium of C57BL/6J mice with and without MI-R injury. (**A**) Fluorescence micrographs showing eYFP-positive hepatic cells (green) in the liver and isolated eYFP-positive hepatic cells in PBS placed on a microscope glass slide covered with a coverslip (note that almost all isolated hepatic cells were eYFP-positive). The length bar is for both images. (**B**) Fluorescence micrographs showing eYFP-positive hepatic cells (green) transplanted into the myocardium of a healthy mouse and into the ischemic myocardium core of mice with MI-R injury. Red: Antibody-labeled cardiac troponin I. Blue: Cell nuclei for both panels A and B. (**C**) Graphic representation of the density of eYFP-positive hepatic cells transplanted to the myocardium of C57BL/6J mice. Means and SDs are presented (n = 6). The solid and open circles represent the means and individual data points.

**Figure 6:**
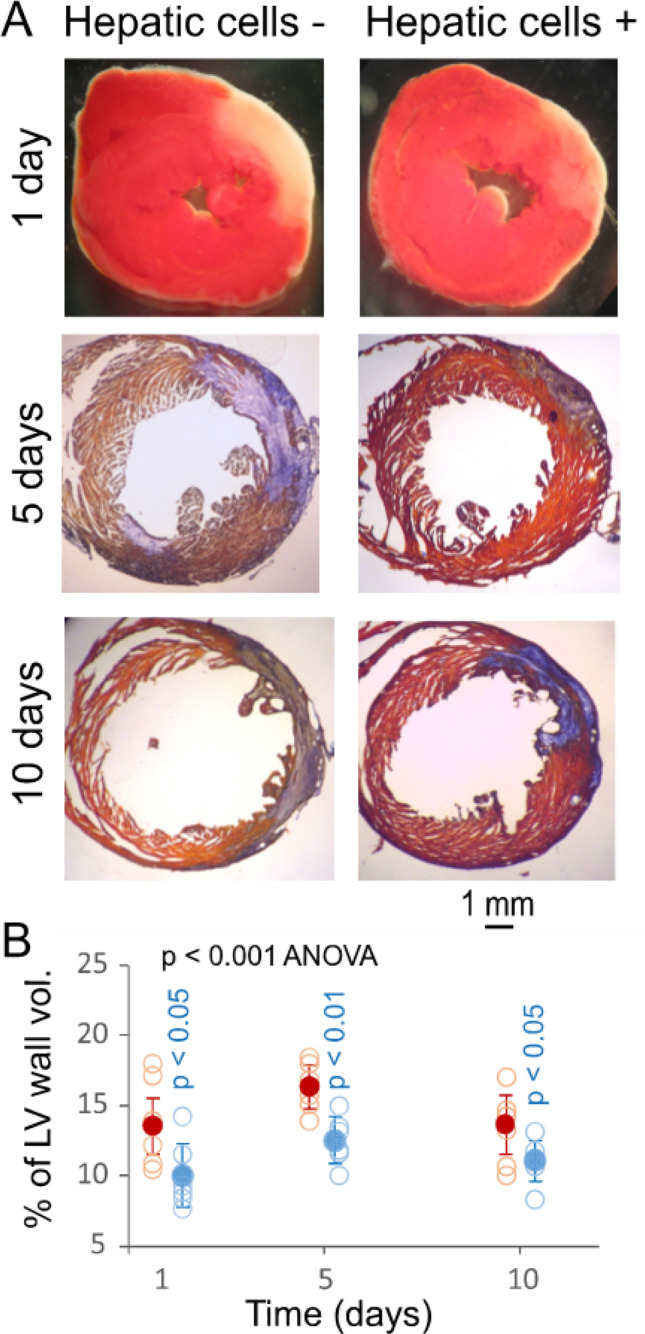
Mitigation of myocardial infarction in response to hepatic cell transplantation. (**A**) Left ventricular (LV) specimens at day 1 of MI-R were treated with TTC for showing acute myocardial infarcts (pale) and LV specimens at day 5 and 10 of MI-R were stained with orange red and methylene blue (AZAN assay) for demonstrating myocardial fibrosis (blue) in mice with and without hepatic cell transplantation. (**B**) Graphic representation of the influence of hepatic cell transplantation on the level of myocardial infarction. Solid red and blue circles: Means ± SDs from individual mice without and with hepatic cell transplantation, respectively. Open red and blue circles: Raw data points (n = 6) from individual mice without and with hepatic cell transplantation, respectively. The blue-colored *p* value at each time point is for comparison between mice with and without hepatic cell transplantation.

To confirm the role of hepatic cells in cardioprotection, hepatic cells were prepared from Alb-Cre/eYFP mice and transplanted into the ischemic myocardium of non-parabiotic C57BL/6J mice immediately following coronary artery ligation. eYFP-positive hepatic cells were present in the ischemic myocardium, although the density of these cells gradually declined (Fig. 5). This treatment mitigated significantly myocardial infarction at 1, 5, and 10 days of MI-R injury (Fig. 6). These observations support the findings from the parabiotic model above and suggest hepatic cell transplantation may serve as a potential cardioprotective strategy against ischemic myocardial injury.

### Hepatic cell-mediated TFF3 delivery to the ischemic myocardium

**Figure 7:**
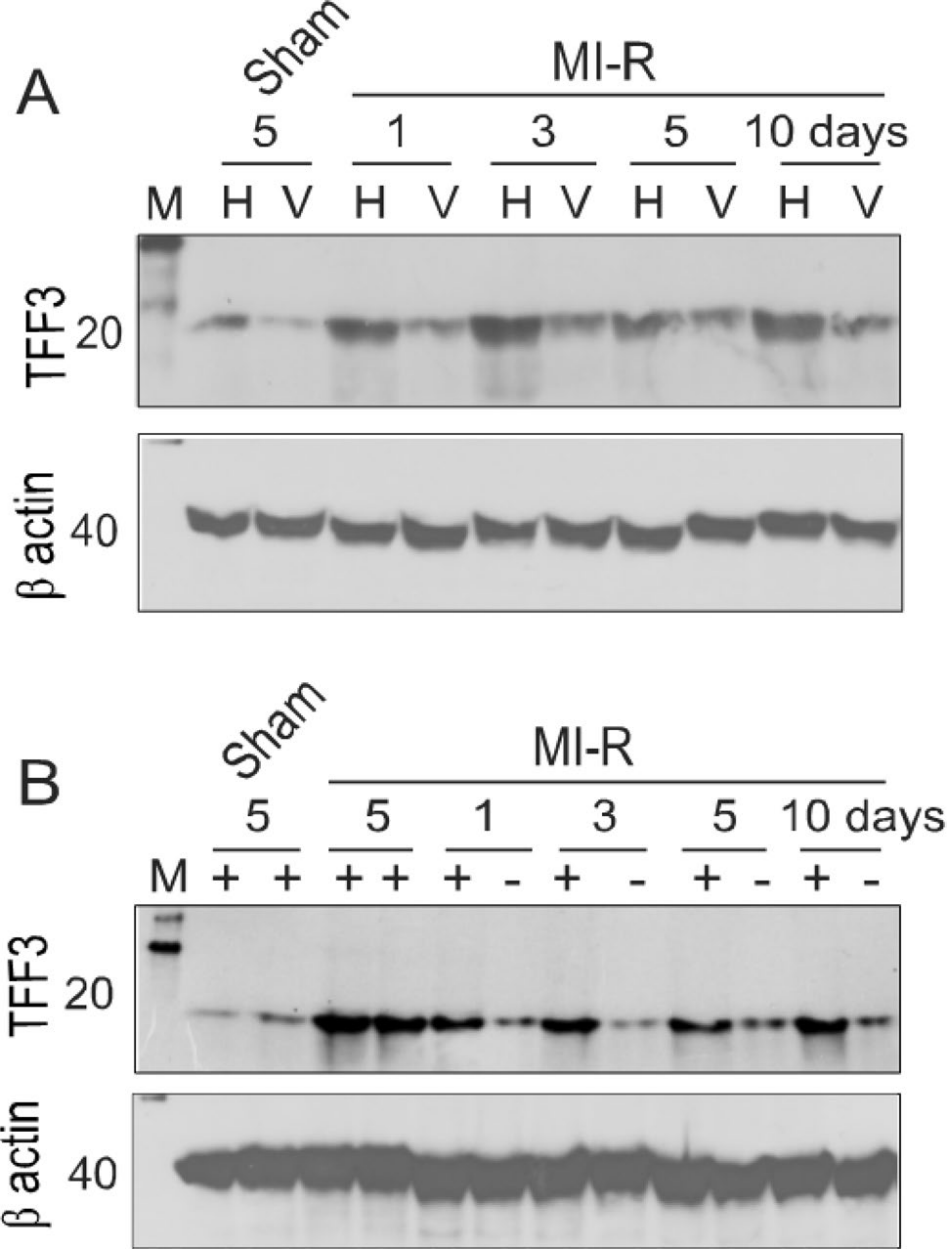
Relative levels of TFF3 in the liver (hepatic cells) and ischemic myocardium. (**A**) Immunoblot analysis of relative TFF3 levels in hepatic cells (H) and the left ventricular myocardium (V) of the liver-intact parabiotic mice with MI-R injury, showing distinct TFF3 levels between the hepatic cells and the ischemic myocardium of the same mouse at each time point. M: Molecular weight markers. (**B**) Immunoblot analysis of relative TFF3 levels in the ischemic myocardium of the liver-intact ( +) and hepatectomized (–) parabiotic mice with bilateral MI-R injury, showing distinct TFF3 levels between the liver-intact and hepatectomized parabiotic mice. For both panels A and B, β actin was used as a loading control. Immunoblot images were from cropped blots. Images from full-length blots are included in the supplementary file.

A fundamental question is how the mobilized hepatic cells protect the myocardium from ischemic injury. Given that the hepatic cells can express TFF3, an endocrine protein capable of mitigating ischemic myocardial injury^23^, one possibility is that the hepatic cells recruited to the ischemic myocardium may release TFF3 to protect the myocardium from ischemic injury. This possibility was evaluated by analyzing the difference in the relative TFF3 level in the ischemic myocardium between the liver-intact and hepatectomized parabiotic mice. In this model, the relative level of TFF3 in the liver of mice with MI-R injury was higher than that in the ischemic myocardium of the same mice (Fig. 7A). The relative level of TFF3 in the ischemic myocardium of the liver-intact parabiotic mice (with a higher density of mobilized hepatic cells) was substantially higher than that in the ischemic myocardium of the hepatectomized parabiotic mice (with a significantly lower density of mobilized hepatic cells) (Fig. 7B). The TFF3 level in the ischemic myocardium of hepatectomized parabiotic mice was comparable to that in the sham control myocardium without MI-R injury (Fig. 7B). Similar levels of TFF3 were detected in the ischemic myocardium of parabiotic mice with bilateral MI-R injury and bilateral hepatic sham operation (without hepatectomy) (Fig. 7B). In cardiac sham-control parabiotic mice, unilateral hepatectomy did not influence the relative level of TFF3 in the myocardium (supplementary Fig. 6). These observations suggest a potential role for the mobilized hepatic cells in the delivery of TFF3 to the ischemic myocardium.

### Role of TFF3 in myocardial protection

**Figure 8:**
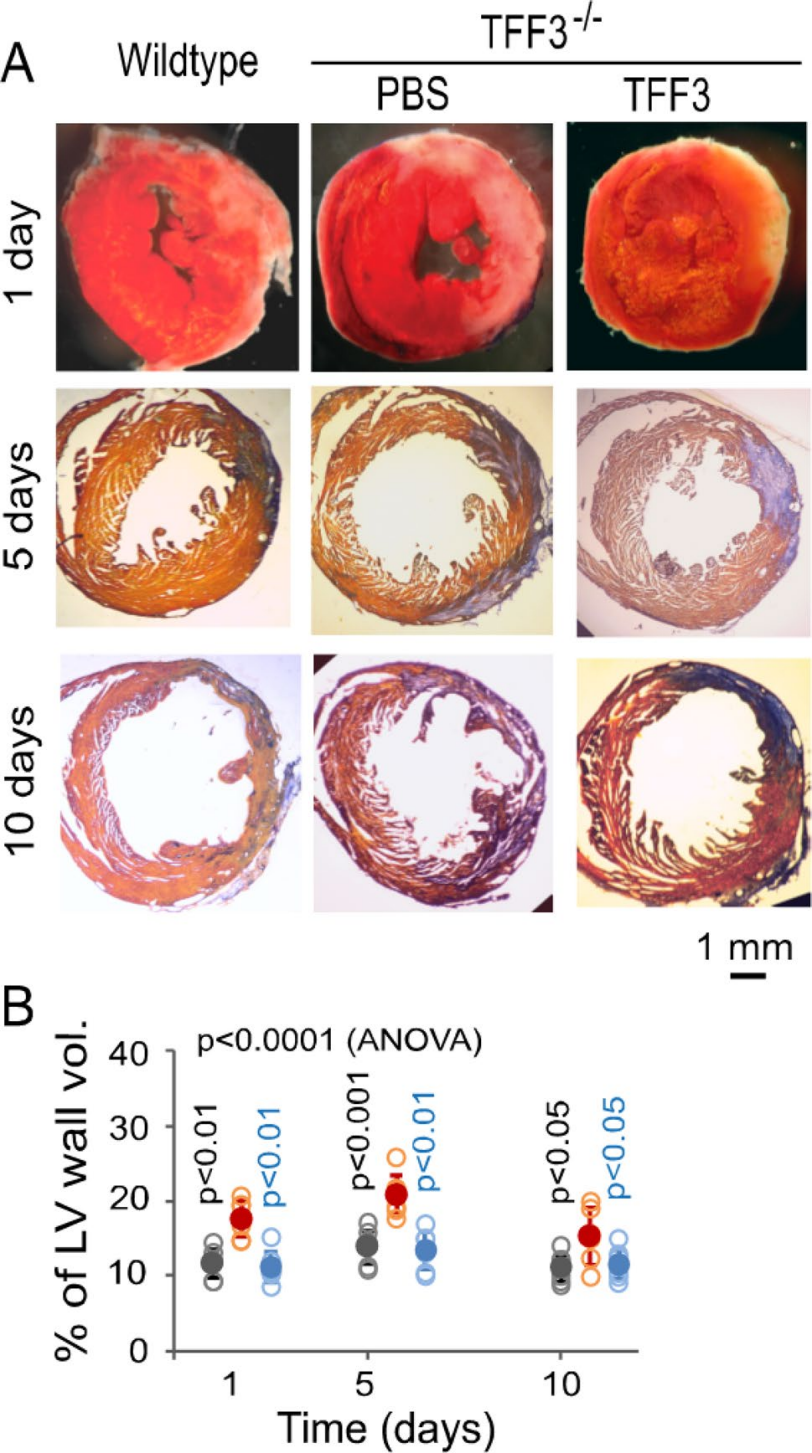
TFF3-mediated mitigation of myocardial infarction. (**A**) Left ventricular specimens treated with TTC for demonstrating acute myocardial infarcts (pale) at day 1 of MI-R injury and stained with orange red and methylene blue (AZAN assay) for showing myocardial fibrosis (blue) at day 5 and 10 of MI-R injury in wildtype and TFF3^−/−^ mice with PBS and recombinant TFF3 administrations into the ischemic myocardium immediately after coronary artery ligation. (**B**) Graphic representation of the influence of TFF3 on the level of myocardial injury. Solid dark gray, red, and blue circles: Means ± SDs from wildtype mice and TFF3^−/−^ mice with PBS and recombinant TFF3 administrations, respectively. Open dark gray, red, and blue circles: Raw data points (n = 6) from individual wildtype mice and TFF3^−/−^ mice with PBS and recombinant TFF3 administrations, respectively. The dark gray-colored p value at each time point is for comparison between the wildtype mice and TFF3^−/−^ mice with PBS administration. The blue-colored *p* value at each time point is for comparison between TFF3^−/−^ mice with PBS and recombinant TFF3 administrations.

To demonstrate the cardioprotective role of TFF3, MI-R injury was introduced to non-parabiotic TFF3^−/−^ mice with wildtype mice as the control. As shown in Fig. 8, the TFF3^−/−^ mice showed significantly higher levels of myocardial infarction at 1, 5, and 10 days after MI-R injury than the wildtype mice. Administration of recombinant TFF3 to the ischemic myocardium of the TFF3^−/−^ mice immediately after coronary artery ligation increased the level of TFF3 in the ischemic myocardium (supplementary Fig. 7) and significantly reduced the level of myocardial infarction in reference to that with PBS administration (Fig. 8). These results confirmed previous observations about the cardioprotective role of TFF3^23^.

## Discussion

### Significance of hepatic cell mobilization in MI-R injury

Myocardial ischemia is a life-threatening condition. Various molecular and cellular systems have evolved to protect the heart from ischemic injury^7,8^. It has long been recognized that there exist regional or paracrine protective mechanisms within the heart, involving cell survival-supporting signaling pathways that can be activated by ischemic cell-released ligands such as adenosine^9–11^, bradykinin^12,13^, opioids^14–16^, vascular endothelial growth factor^19,20^, and follistatin-like protein 1^30,31^. The present investigation has identified an additional cardioprotective mechanism – mobilization of hepatic cells to the ischemic myocardium to deliver TFF3, a secreted protein capable of supporting myocardial survival. Although the mechanisms of TFF3 action remain to be investigated, this discovery suggests the presence of a liver-based distant process for protection against myocardial injury under an ischemic attack. In addition to the liver, the bone marrow and spleen can discharge hematopoietic cells into the circulatory system to aid in cardioprotection in response to ischemic myocardial injury^32–37^. It seems that the distant mechanisms are an integral part of the cardioprotective system, acting together with the regional cardioprotective mechanisms to augment myocardial survival under an ischemic insult. The involvement of multiple organs in cardioprotection conforms to the labor-division mechanism in multi-celled organisms—the collaborative action of multiple cell types or organs to help implement or support a function designated to a selected cell type or organ^8^. In ischemic myocardial injury, a cardioprotective labor-division mechanism reduces the burden to the injured cardiac cells that are incapable of implementing all designated functions, including protective gene expression, and helps to prevent heart failure.

Hepatic cell mobilization-mediated TFF3 delivery to the ischemic myocardium represents an example supporting the cardioprotective labor-division mechanism. Cardiac cells express a substantially lower level of TFF3 compared with hepatic cells under sham control and ischemic conditions as shown in this investigation. The mitigation of myocardial injury in response to hepatic cell mobilization-induced elevation in the myocardial TFF3 level suggests the necessity of endocrine TFF3 for cardioprotection. TFF3 has been originally found in the intestinal epithelial cells and known as a protective factor against intestinal epithelial injury under healthy and pathological conditions^24–29^. TFF3 is also upregulated and released from the liver to protect the brain from injury in response to experimental ischemic stroke^38^. Thus, TFF3 serves potentially as a protective factor for multiple organ systems. The delivery of TFF3 to the ischemic myocardium by hepatic cell mobilization may represent a more effective approach for reaching a critical cardioprotective level than TFF3 delivery from the liver through the circulatory system.

### The necessity of the parabiotic model

Mouse parabiosis, the surgical union of two mice through a skin connection to establish cross-animal circulatory communications, was used as a model in this investigation to demonstrate the cardioprotective action of the mobilized hepatic cells. Although parabiosis adds another layer of complexity to the MI-R injury model, it is necessary to use such a model to establish loss- and gain-of-hepatic cell mobilization conditions. A simple, effective approach to eliminate hepatic cell mobilization in an animal model is to remove the liver; however, such an approach causes rapid animal death. A parabiotic model allows the removal of a single liver from a parabiotic pair. The remaining liver is sufficient to support both mice. This modification establishes a condition with hepatic cell mobilization in the liver-intact mouse. As the mobilized hepatic cells are eliminated through the aorta and cannot reach the circulatory system of the parabiotic partner mouse with hepatectomy, hepatic cells can only be recruited to the heart of the liver-intact mouse, but not to the heart of the hepatectomized mouse, establishing concurrent gain- and loss-of-hepatic cell conditions in the same parabiotic pair. Thus, this model can be used to evaluate the role of the mobilized hepatic cells in protection against ischemic myocardial injury.

### Regulation of hepatic cell mobilization

Hepatic cell mobilization is regulated by endocrine signaling mechanisms activated in response to MI-R injury, involving interleukin (IL) 6 released primarily from leukocytes recruited to the ischemic myocardium^22^. IL6, a major inflammatory regulatory factor^39^, can enter the circulatory system to activate leukocytes, enabling leukocytes to extravasate to the hepatic parenchyma^22^. The liver-retained leukocytes can express and release MMP2, a proteinase capable of degrading the collagen matrix. These activities cause dissociation of hepatic cells nearby the liver-retained leukocytes^22^. The dissociated hepatic cells can be discharged into the circulatory system. An interesting observation was that the hepatic cells immediately mobilized to the hepatic central veins are often clustered with leukocytes, which dissociate from the mobilized hepatic cells in the vena cava under the influence of blood flow^22^. These observations support the presence of systems signaling mechanisms between the heart and liver for the activation of distant cardioprotective processes in response to ischemic myocardial injury.

### The fate of the mobilized hepatic cells

Hepatic cell mobilization is a transient process, occurring primarily within 10 days after MI-R injury. At the observation time points 1, 3, 5, and 10 days after MI-R injury, the mobilized hepatic cells were present in the left ventricular chamber and the ascending aorta, but absent in the peripheral arteries and veins. Although it is unclear what causes the elimination of the mobilized hepatic cells within the aorta, it is possible to speculate that blood flow-associated fluid shear stress may play a role. The aortic fluid shear stress, the highest through the longest large blood vessel, may cause disintegration of the mobilized hepatic cells, a cell type not subject to a high fluid shear stress environment under physiological conditions and thus susceptible to mechanical injury. However, this speculation requires experimental verification. As hepatic cells are not circulatory cells, their rapid clearance prevents hepatic cell accumulation in the circulatory system. The hepatic cells recruited to the ischemic myocardium were present up to 10 days. The population of hepatic cells in the ischemic myocardium reached a peak at day 5 and returned toward the sham-control level at day 10. This time course was coincident with that of acute ischemic myocardial injury. These observations suggest that hepatic cell mobilization is induced for myocardial protection in response to acute ischemic injury. However, the fate of the hepatic cells recruited to the ischemic myocardium remains to be investigated.

### Regulation of TFF3 expression

An important question is how the heart communicates with the liver in MI-R injury to cause TFF3 expression. A previous report has suggested that the cytokine IL6 may play a role in stimulating TFF3 expression in the liver^22^. IL6 is upregulated and released from leukocytes recruited to the ischemic myocardium and can enter the circulatory system^22^. This cytokine may stimulate hepatic cell expression of TFF3 as the mouse TFF3 gene promoter/enhancer region contains binding sites for STAT3^21^, a transcription factor mediating IL6-induced gene expression^40,41^. Indeed, IL6 has been shown to stimulate TFF3 expression via the mediation of STAT3 in gastrointestinal epithelial cells^40^ and biliary epithelial cells^41,42^. Although IL6 causes TFF3 expression, this cytokine was not used to stimulate TFF3 expression in this investigation because IL6 itself is a cardioprotective factor and can activate various inflammatory responses that complicate data interpretation for the cardioprotective action of TFF3.

### Technical concerns

There are several technical concerns for this investigation. The mechanisms of TFF3 action remain poorly understood. A TFF3 receptor and associated signaling pathway(s) have not been identified in cardiomyocytes. Given the cardioprotective role of TFF3, it would be interesting to study the signaling mechanisms of TFF3 in ischemic myocardial injury. The Evans blue-based measurement of the area-at-risk is often conducted and used as a reference to evaluate the relative level of acute myocardial infarction identified by the TTC assay. This approach was not used in this investigation because two different assays were used to evaluate myocardial infarction—the TTC assay for 1- and 3-day analyses and the AZAN assay for 5- and 10-day analyses. It is difficult to measure accurately the area-at-risk by the Evans blue assay at 5 and 10 days of MI-R injury because of the presence of newly generated blood vessels that perfuse the ischemic myocardium, resulting in false negative estimation of the area-at-risk. The TTC assay, designed to detect live and dead cells, was not used for the 5- and 10-day analysis of myocardial infarction because of the presence of leukocytes and regenerated fibroblasts in the ischemic myocardium, causing false negative measurement of myocardial infarction. The AZAN assay, designed to detect the infarcted myocardium based on the presence of fibrotic tissue, is not suitable for identifying acute myocardial infarction at 1 and 3 days prior to fibrosis. Given these technical limitations, it is difficult to use either the TTC or AZAN assay for all observation time points. To establish a common basis for comparison in myocardial infarction by using both TTC and AZAN assays, coronary artery ligation was conducted at the same location—the second diagonal branch bifurcation point for all mice tested at all time points. This location was consistent among mice tested in this investigation in terms of the relative distance of the ligation site to the cardiac apex.

## Concluding remarks

Ischemic myocardial injury has long been known to activate regional signaling mechanisms that protect the cardiomyocyte from death. This investigation demonstrated that the liver was able to respond to MI-R injury to mobilize its cells to the circulatory system and ischemic myocardium for mitigating myocardial infarction by releasing the cardioprotective factor TFF3. These findings suggest the presence of distant cardioprotective mechanisms that act in concert with the regional mechanisms to augment myocardial survival under an ischemic attack.

## Materials and methods

### Animal models

Alb-Cre/eYFP, C57BL/6J, TFF3^−/−^, and 129S1/SvImJ mice, 2-month old and male, were used in this investigation. The Alb-Cre/eYFP mice were used to induce the expression of enhanced yellow/green fluorescence protein (eYFP) in hepatic cells, including hepatocytes and bile ductular epithelial cells, allowing the identification of hepatic cells mobilized to the circulatory system and ischemic myocardium^22^. These mice were generated by crossing the mouse strain B6.Cg-Tg(Alb-cre)21Mgn/J (C57BL/6J background, Jackson Laboratory) expressing the Alb-Cre gene (albumin gene promoter-driven Cre recombinase gene) with the mouse strain B6.129X1-Gt(ROSA)26-Sor^tm1(EYFP)Cos^/J (C57BL/6J background, Jackson Laboratory) expressing the eYFP gene controlled by a loxP-flanked stop sequence that blocks eYFP expression in the lack of Cre recombinase. The Cre recombinase, specifically expressed in the liver driven by the albumin gene promoter, can delete the stop sequence for the eYFP gene between the loxP sites, resulting in hepatic cell-specific expression of eYFP. This model was used to identify hepatic cells mobilized to the circulatory system and ischemic myocardium in MI-R injury by fluorescence microscopy and immunohistochemistry with an anti-eYFP antibody (Clontech). C57BL/6J mice were used as a control. The TFF3^−/−^ mice (129;B6-Tff3^tm1Dkpy^/J, 129S1/SvImJ background, Jackson Laboratory) were used to evaluate the cardioprotective role of TFF3 in MI-R injury with 129S1/SvImJ mice as a control. Experimental procedures were approved by the Northwestern University Animal Care and Use Committee. The study on mice was carried out in compliance with the “Guide for the Care and Use of Laboratory Animals” by the National Research Council, USA and the guidelines of ARRIVE (Animal Research: Reporting of In Vivo Experiments).

### Myocardial ischemia–reperfusion injury

Myocardial ischemia–reperfusion (MI-R) injury was induced in mice by 30-min ligation of the left anterior descending coronary artery at the second diagonal branching point as described^23,43^. Gender- and body weight-matched mice with sham operation were used as controls. The animals were examined at 1, 3, 5, and 10 days. These time points were selected based on the time course of hepatic cell mobilization in response to ischemic myocardial injury^22^. The sample size of each group was determined by power analysis.

### Parabiosis

A parabiotic mouse model was established by connecting the lateral skins of two male, 2-month old, size-matched Alb-Cre/eYFP mice from the same litter. In this model, blood flow communication can be established between the two parabiotic mice within 2 weeks^44,45^. This model was used to introduce concurrent gain- and loss-of-hepatic cell mobilization conditions to the two parabiotic mice by unilateral hepatectomy (removing the liver of one parabiotic mouse) and bilateral MI-R injury, allowing the evaluation of the cardioprotective action of the mobilized hepatic cells. MI-R injury was introduced to both mice of each parabiotic pair at 2 weeks after parabiotic surgery. Immediately following coronary artery ligation, hepatectomy was carried out in one of the two parabiotic mice to remove > 90% of the liver (note that it was difficult to remove completely the liver because of the short distance between the liver and the vena cava), while a hepatic sham operation was performed in the other parabiotic mouse. In this model, the mobilized hepatic cells, while able to reach the heart of the liver-intact mouse, were unable to reach the hepatectomized mouse as these cells were rapidly eliminated in the aorta, establishing gain- and loss-of-hepatic cell mobilization conditions in the same parabiotic pair. Cardiac sham operation was introduced to parabiotic mice with unilateral hepatectomy. Left ventricular specimens were examined and used as controls at 1 and 5 days. In addition, Alb-Cre/eYFP and C57BL/6J mice were paired to establish a parabiotic model for testing hepatic cell exchange between the two mice of each parabiotic pair.

### Hepatic cell isolation and transplantation

To confirm the cardioprotective role of hepatic cells, eYFP-positive hepatic cells were isolated from Alb-Cre/eYFP mice and transplanted to the ischemic myocardium of C57BL/6J mice. To isolated hepatic cells, collagenase (0.25% in PBS) was introduce to the liver of Alb-Cre/eYFP mice by abdominal aorta perfusion (37 °C, 15 min after deep anesthesia) to cause hepatic cell dissociation. Total cells were collected from the collagenase-treated liver and centrifuged in a Percoll medium for isolating hepatic cells^23,46^. The purity of eYFP-positive hepatic cells was assessed by fluorescence microscopy. The isolated hepatic cells (~ 2 × 10^5^ in 20 µl PBS) were immediately used and injected slowly to 4 locations of the ischemic myocardium of a body weight- and gender-matched C57BL/6J mouse following coronary artery ligation. C57BL/6 J mice without hepatic cell transplantation were used as a control. At 1, 5, and 10 days following hepatic cell transplantation, myocardial specimens were collected and cut into cryo-sections of 5 μm in thickness. The transplanted eYFP hepatic cells in myocardial specimen sections were visualized by fluorescence microscopy. The density of these cells (per unit area) in the myocardial specimen sections was measured at 1, 5, and 10 days following myocardial ischemia–reperfusion injury. The level of myocardial infarction was analyzed at these time points with 6 mice each time point as describe below.

### Recombinant TFF3 administration

To test the role of TFF3 in cardioprotection, recombinant TFF3 (CYT-781, ProSpec) 50 ng in 20 µl PBS was injected slowly to 4 locations of the ischemic myocardium of a TFF3^−/−^ mouse immediately after coronary artery ligation. TFF3^−/−^ mice with PBS injection were used as a control. The level of myocardial infarction was analyzed at 1, 5, and 10 days with 6 mice each time point as described below.

### Left ventricular dp/dt and echocardiography

The contractile function of the left ventricle was evaluated based on left ventricular fractional shortening by echocardiography and left ventricular dp/dt by analyzing the first derivative of blood pressure. A SonoScape ultrasound system and a Millar micro-catheter pressure transducer system were used for these measurements, respectively, as described^23,43^.

### Microscopy

Blood samples of 20 µl each were collected from the left ventricular chamber, ascending aorta, femoral artery, and femoral vein of each anesthetized mouse, smeared on glass slides, treated with Hoechst 33,258 for staining cell nuclei, and used to count EYFP-positive cells by fluorescence microscopy. The relative density of EYFP-positive cells was calculated in reference to the total number of cell nuclei. The heart of a fully anesthetized mouse was perfused with 4% formaldehyde in PBS for 30 min, cut into cryo-sections of 10 µm in thickness, and tested by fluorescence microscopy to identify EYFP-positive cells. The same blood and cardiac specimens were also tested by immunohistochemistry with a primary anti-EYFP antibody and a horseradish peroxidase-conjugated secondary antibody to confirm the identity of the fluorescence microscopy-detected EYFP-positive cells. There are two hepatic cell types that express EYFP – hepatocytes and bile ductular cells. The bile ductular epithelial cells were identified based on co-expression of EYFP and cytokine 19 (CK19), a bile ductular cell-specific marker within the liver.

### Assessment of myocardial infarction

The level of myocardial infarction was assessed by using the TTC assay at 1 and 3 days and AZAN assay at 5 and 10 days. The TTC assay was not used at 5 and 10 days because of the presence of leukocytes and fibroblasts that show positive (red) TTC staining in the infarcted myocardium. The AZAN assay was used at 5 and 10 days to stain fibrotic tissue. For the TTC assay, the heart was removed rapidly from a euthanized mouse, frozen at − 90 °C for 5 min, and cut into 0.5 mm-thick serial slices, stained with 1% TTC in PBS at 37 °C for 30 min, and examined by microscopy. For the AZAN assay, the heart was perfused with 4% formaldehyde in PBS for 30 min, cut into 20 µm-thick serial sections by using a cryo-microtome, and stained with AZAN solutions as described^23,43^. The fraction of myocardial infarcts was measured in both TTC and AZAN assays in reference to the left ventricular wall volume.

### TFF3 expression

The relative expression of TFF3 was assessed in the ischemic myocardium and hepatocytes by immunoprecipitation and immunoblot analyses as described^38^. Hepatic cells were isolated by collagenase treatment and Percoll-mediated centrifugation as described^23^.

### Statistics

Means and standard deviations were calculated for all measured parameters. The Student’s t-test was used for comparisons between two groups. The analysis of variance (ANOVA) test was used for comparisons between multiple groups. Post-hoc comparisons were carried out by using the Bonferroni method. Sample sizes were estimated by power analysis at the power level of 0.8 and α level of 0.05. A difference was considered statistically significant at *p* < 0.05.

## Supplementary Information

Supplementary Figures and Tables.
