# Supplementary material for: Hepatic cell mobilization for protection against ischemic myocardial injury
Source: Sci Rep. 2021 Aug 4;11:15830. doi: 10.1038/s41598-021-94170-z (PMC8339068; doi:10.1038/s41598-021-94170-z)
Supplement: Supplementary file 1 — Supplementary Figures and Tables. [file 41598_2021_94170_MOESM1_ESM.docx]

**Hepatic cell mobilization for protection against ischemic myocardial injury**

Shu Q. Liu^1§^, John B. Troy^1^, Chi-Hao Luan^2^, Roger J. Guillory^1*^

1. Biomedical Engineering Department, Northwestern University, Evanston, IL 60208, USA
2. High Throughput Analysis Laboratory, Northwestern University, Evanston, IL 60208, USA

§ Author for correspondence [sliu@northwestern.edu](mailto:sliu@northwestern.edu)

* Current address: Department of Biomedical Engineering, Michigan Technological University, Houghton, MI 49931, USA

**Supplementary Figures**

**Figure S1**. Immunofluorescence micrographs showing the lack of eYFP-positive cells in the intact myocardium (remote to the ischemic region) of liver-intact and hepatectomized parabiotic Alb-Cre/eYFP mice with MI-R injury.


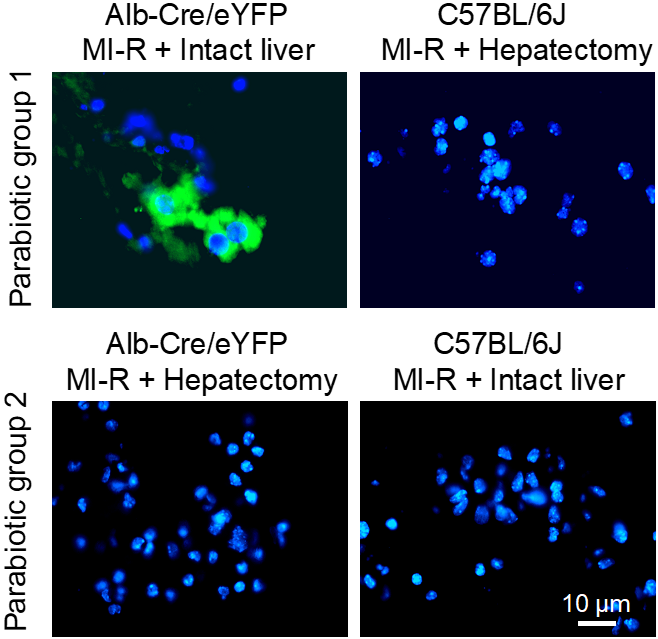


**Figure S2**. Fluorescence micrographs showing the lack of hepatic cell exchanges between the circulatory systems of the two mice of a parabiotic pair, which comprised an Alb-Cre/eYFP mouse and a C57BL/6J mouse with bilateral MI-R injury and alternated unilateral hepatectomy. No eYFP-positive hepatic cells were found in the circulatory system of the C57BL/6J parabiotic mouse that experienced MI-R injury and hepatectomy, when the Alb-Cre/eYFP partner mouse experiences MI-R injury without hepatectomy. Few eYFP-positive hepatic cells were present in the circulatory systems of both parabiotic mice when the Alb-Cre/eYFP partner mouse experienced MI-R injury and hepatectomy, and the C57BL/6J parabiotic mouse experienced MI-R injury without hepatectomy.

**Figure S3.** Graphic representation of the fraction of circulating eYFP-positive cells in reference to the total nucleated cells from the ascending aorta (A. aorta), femoral artery (FA), and femoral vein (FV) of Alb-Cre/eYFP mice with 5-day ischemic myocardial injury. Means and standard deviations are presented (n = 6). The p value on each column is for comparison between the ascending aorta and FA or FV.

**Figure S4**. Left ventricular specimens from liver-intact and hepatectomized parabiotic mice with cardiac sham operation at 1 day (TTC assay) and 5 days (AZAN assay). The length scale is for all panels.

**Figure S5**. Graphic representation of the left ventricular (LV) wall thickness from liver-intact and hepatectomized parabiotic mice with bilateral MI-R injury by echocardiography. Solid blue and red circles: Means +/- SDs from liver-intact and hepatectomized parabiotic mice, respectively. Open red and blue circles: Raw data points (n = 6) from liver-intact and hepatectomized parabiotic mice, respectively.

**Figure S6**. Immunoblot analysis of relative TFF3 expression in the ischemic myocardium of parabiotic cardiac sham-control mice with and without unilateral hepatectomy. L+: Liver intact. L-: Hepatectomy. β actin was used as a loading control. Immunoblot images were from cropped blots. Images from full-length blots are shown below in this supplementary file.


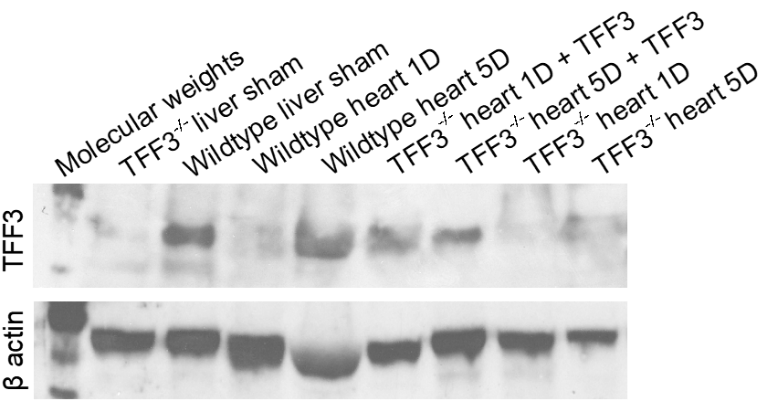


**Figure S7**. Immunoblot analysis showing the relative levels of TFF3 in the wildtype and TFF3^-/-^ liver, in the myocardium of wildtype mice with 1- and 5-day MI-R injury, and in the myocardium of TFF3^-/-^ mice with 1- and 5-day MI-R injury with and without administration of recombinant TFF3 (+ TFF3). Immunoblot images were from cropped blots. Images from full-length blots are shown below in this supplementary file.

**Unedited gel images**

Uncropped immunoblot images for Figure 7.

Uncropped immunoblot images for supplementary Figure S6.


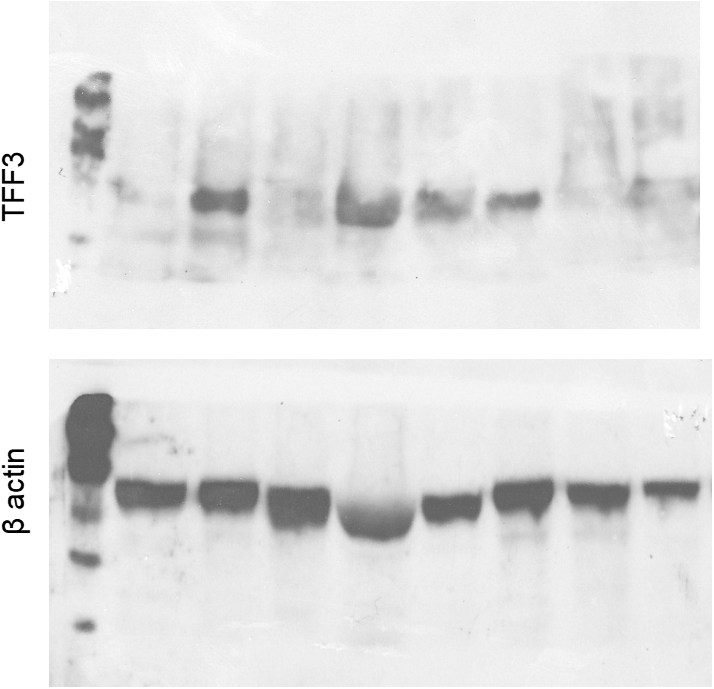


Uncropped immunoblot images for supplementary Figure S7.

**Supplementary Table 1.** Numbers of eYFP-positive cells in blood samples from the left ventricular (LV) chamber and ischemic myocardium of Alb-Cre/eYFP parabiotic mice with intact liver and hepatectomy in 5-day MI-R injury.

Time Intact liver Hepatectomy

(days) LV chamber Ischemic myocardium LV chamber Ischemic myocardium

eYFP-positive cells ∕µl eYFP-positive cells ∕mm^2^ eYFP-positive cells ∕µl eYFP-positive cells ∕mm^2^

(Means +/- SDs) (Means +/- SDs) (Means +/- SDs) (Means +/- SDs)

1 14 +/- 6 23 +/- 12 0 +/- 0 0 +/- 0

3 35 +/- 11 105 +/- 30 7 +/- 6 13 +/- 11

5 72 +/- 6 270 +/- 46 13 +/- 6 33 +/- 14

10 13 +/- 5 80 +/- 30 3 +/- 4 8 +/- 8

**Supplementary Table 2**. CD45-positive cells in the ischemic myocardium of mice with intact liver and hepatectomy at 5 days of MI-R injury.

Intact liver Hepatectomy

CD45+ cells **∕** Total cell nuclei (%) CD45+ cells **∕** Total cell nuclei (%) p (t-test)

20.8 +/- 2.2% 19.7 +/- 1.9% > 0.2

**Supplementary Table 3**. CK19-positive and eYFP-positive cells recruited to the ischemic myocardium of mice with intact liver at 5 days of MI-R injury.

eYFP+ cells **∕** Total cell nuclei CK19+ eYFP+ cells **∕** eYFP+ cells CK19+ eYFP ̶ **∕** Total cell nuclei

(%) (%) (%)

2.7 +/- 0.5% 23 +/- 9% 0.15 +/- 0.11%

eYFP+: eYFP-positive; eYFP ̶ : eYFP-negative; CK19+: CK19-positive; CD45+: CD45-positive.

**Supplementary Table 4.** Numbers of eYFP-positive cells in the circulatory systems of Alb-Cre/eYFP – C57BL/6J-paired parabiotic mice with bilateral MI-R injury (5 days) and alternated unilateral hepatectomy between the two parabiotic mice, showing the lack of hepatic cell exchange between the two parabiotic mice.

Parabiotic group 1 (5-day MI-R) Parabiotic group 2 (5-day MI-R)

Alb-Cre/eYFP intact liver C57BL/6J hepatectomy Alb-Cre/eYFP hepatectomy C57BL/6J intact liver

eYFP-positive cells ∕µl eYFP-positive cells ∕µl eYFP-positive cells ∕µl eYFP-positive cells ∕µl

(Means +/- SDs) (Means +/- SDs) (Means +/- SDs) (Means +/- SDs)

76 +/- 12 0 +/- 0 11 +/- 8 0 +/- 0
